# Preoperative antithrombotic use is associated with delayed mobilization and plural blood transfusions following hip fracture surgery: a case–control study

**DOI:** 10.1007/s41999-026-01581-0

**Published:** 2026-08-25

**Authors:** Henrik Nupponen, Hanna Pajulammi, Anssi Korppi, Esa Jämsen

**Affiliations:** 1 Faculty of Medicine and Health Technology, Tampere University, Tampere, Finland; 2 Central Finland Health Care District, Central Finland Hospital Nova, Jyväskylä, Finland; 3 Department of Anesthesia, Tampere University Hospital, Tampere, Finland; 4 Faculty of Medicine (Clinicum), University of Helsinki, Helsinki, Finland; 5 Department of Geriatrics, Helsinki University Hospital, Helsinki, Finland

**Keywords:** Hip fracture, Anticoagulants, Antithrombotics, Post-operative mobilization

## Abstract

**Purpose:**

Early surgical treatment is important to allow a prompt start for rehabilitation after hip fracture. In patients using antithrombotic drugs, the surgery is often delayed, but it is unclear how this affects early post-operative recovery.

**Methods:**

The course of treatment and early post-operative outcomes of hip fracture patients, aged ≥ 70 years, using either warfarin, direct oral anticoagulants (DOACs), or adenosine diphosphate. (ADP) inhibitors were compared to age- and sex-matched controls not using such drugs, operated during the same observation period at the same hospital. All data were retrospectively collected from electronic medical records.

**Results:**

306 patients (53% of all operations; mean age 87 years, 63% females) were included. Patients using DOACs or ADP inhibitors had their operation later than controls and warfarin users (2 and 2.5 days, respectively, compared to 1 day after admission; *p* < 0.001). Antithrombotic use was also associated with later mobilization (cases 2nd vs. controls 1st post-operative day; *p* = 0.004) and greater use of red blood cell (RBC) transfusions (14% of warfarin, 26% of DOAC, and 10% of ADP inhibitor users received ≥ 2 units of RBCs, compared to 5% of controls; *p* = 0.003). After adjustment for comorbidities, cognition, mobility, and disability, antithrombotic use was associated with RBC transfusions and use of DOACs with delayed surgery and RBC transfusions. There were no differences in 30-day mortality.

**Conclusion:**

Preoperative use of antithrombotic drugs may delay mobilization and increase the need for RBC transfusions after hip fracture surgery. Delayed surgery and mobilization may lead to worse rehabilitation outcome.

## Key summary points

**Aim:**

To assess the effects of anticoagulant use on the treatment of hip fracture and post-operative rehabilitation in geriatric population.

**Findings:**

The use of antithrombotics was associated with delayed mobilization and the need for plural blood transfusions. Direct oral anticoagulants and ADP inhibitors were also associated with delayed surgery and longer length of stay.

**Message:**

Antithrombotics may delay hip fracture management, highlighting the importance of enabling early mobilization for optimal functional outcomes.

## Introduction

Hip fracture is a major fall-related injury in older people, often associated with decrease of functional ability and increased mortality. Only half of the patients who lived independently before the fracture recover to the same performance level [1] and one-fifth of those receiving home care end up in long-term care within one year of the fracture [2].

The factors affecting recovery have been under intensive research. In earlier studies, operation within 48 h (lower mortality) early [3, 4], mobilization (better mobility function, lower mortality and fewer complications) [5, 6], and early removal of indwelling urinary catheter (IUC) (lower risk of decline of mobility level) [2] have been associated with improved outcomes, emphasizing the importance of prompt management of the fracture. Use of antithrombotic drugs, however, may lead to delaying the surgery due to risks of bleeding complications [7, 8]. Over the last decade, use of direct oral anticoagulants (DOACs) and ADP inhibitors has increased rapidly, bringing up challenges to surgery and anesthesia, as the effects of these drugs cannot be reversed (dabigatran being an exception).

In most previous studies, the surgery has been performed later in antithrombotic users [7, 9–15] whereas results concerning the need for red blood cell (RBC) transfusions have been mixed [8, 10, 12, 16, 17]. To the best of our knowledge, there are no earlier studies about the effects of antithrombotic drugs on post-operative recovery. Moreover, the previous studies lack information about pre-fracture functional ability, living arrangements, and cognition that may all affect the course of treatment as well as the outcomes [18, 19].

This case–control study aimed to evaluate how antithrombotic use affects the course of treatment and early functional outcomes after hip fracture, while accounting for baseline variables.

## Materials and methods

The patients were identified from hospital discharge records using ICD-10 diagnosis codes (S72.0, S72.1, and S72.2). Patients using antithrombotics, including warfarin, direct oral anticoagulants (DOACs), and/or ADP inhibitors at the time of admission, were identified from the electronic medical records and they represent the cases. Each case was matched with one control patient not using antithrombotics based on sex and the following criteria: 1) age within 5 years of the age of the case (if there were several candidates, the one with smallest age gap was chosen), 2) day operation within 7 days of the case’s operation, and 3) same fracture type. If a control patient fulfilling all the criteria could not be found, persons with other fracture type and greater difference in the day of operation were also accepted. All cases had a control with the same sex and age ± 5 years, and 69% had a control matching all three criteria. In 44 cases, the difference in the date of surgery was > 7 days (8–48 days). Fracture type differed in 54 cases but with no difference between the groups (Table 1). Two case patients had two hip fractures during the observation period. Both of their fractures were included and had separate controls matched using the above-mentioned criteria. Use of acetylsalicylic acid was allowed in both study groups. Patients with atrial fibrillation without antithrombotic medication or uncertainty of antithrombotic use were excluded.

**Table 1:** Demographic and preoperative characteristics of cases and controls

	Cases (*n* = 153)	Controls (*n* = 153)	*P* value
Age	87* (71–101)	87* (71–97)	0.674
Female sex	63%	63%	1.000
Living arrangement**			0.580
Home	74%	71%	
Other (residential care, institution)	26%	29%	
Walking aid			0.008
None	22%	35%	
Cane or forearm crutches	12%	15%	
Walker	57%	36%	
Wheelchair	3%	3%	
Unknown	7%	11%	
Performance in daily activities			0.012
Fully independent	24%	33%	
Needs help in activities outside home	14%	18%	
Needs help in housekeeping	33%	16%	
Needs help in personal activities of daily living	22%	27%	
Unknown	7%	7%	
Number of chronic diseases	4* (1–9)	3* (0–7)	< 0.001
Number of regular medications	8* (2–16)	6* (0–18)	< 0.001
Memory disorders***	50%	58%	0.151
CHA_2_DS_2_-VASc	5* (2–8)	4* (1–8)	0.001
HAS-BLED	2* (1–4)	2* (1–5)	0.133
Fracture type			0.203
Femoral neck	53%	63%	
Pertrochanteric	37%	30%	
Subtrochanteric	10%	7%	

^*^Median (range), ** Information missing about living arrangement from 5 patients,

^***^Diagnosed dementia or obvious memory problems without a specific diagnosis

Patient data were collected from multiple sources in the electronic medical records of the university hospital. In patients residing in the largest city of the area, medical records of the adjacent secondary care orthogeriatric ward were also reviewed. Background data were retrieved from the physicians’ notes and information about operation-related variables from anesthesiology records. The number of diseases was calculated based on the chronic conditions listed in physicians’ notes in medical charts. Details of post-operative care and progression of recovery (mobilization and removal of IUC) were extracted from nurses’ daily notes and discharge abstract. Medications were recorded from the electronic medication list and physicians’ notes. CHA_2_DS_2_-VASc (risk of thromboembolism in atrial fibrillation) and HAS-BLED (risk of bleeding) scores [20] were calculated for both cases and controls.

During the study period, one of the four orthopedic operation rooms was dedicated to hip fractures. Decision of patients’ operating order was made daily in meeting with organizing orthopedist, anesthesiologist, nurse and ward bed manager. The patient to be operated first on the next day was chosen in a similar meeting later during the day. An anesthesiologist was consulted on anesthesia modality and surgical risks, but the organizing orthopedist decided on operating order. Factors that affected the operating order were e.g., use of ADP inhibitors or DOACs. Warfarin users were given vitamin K at emergency department and additional prothrombin complex if INR was > 1,7. RBC transfusions were given perioperatively if hemoglobin was < 70 g/L (< 90 g/L in patients with severe heart disease). Peripheral femoral blocks were not used routinely preoperatively or at the wards. Following national guideline, immediate full weight-bearing was typically allowed.

### Outcomes

The following outcomes were compared between the cases and the controls: time from admission to surgery (TTS), duration of surgery, length of stay at postanesthesia care unit (PACU), blood loss during surgery, length of total and post-operative stay at the operating hospital, changes in hemoglobin levels during hospital stay, use of RBC transfusions, time to mobilization (on which day postoperatively the patient took at least one step [walking aids and support were allowed], and if the patient was mobilized before discharge from the operating hospital), time to IUC removal, and 30-day mortality. In addition, for patients whose care could be followed at the secondary care orthogeriatric ward, the total length of stay, including stay both in the operating hospital and at the secondary care orthogeriatric ward, was calculated.

### Statistics

At first, the comparisons were made between the cases and the controls. Then, cases were divided into three groups based on type of antithrombotic use at the time of hip fracture (warfarin, DOACs, ADP inhibitors) and these groups were compared to the control group.

Analyses were made with SPSS (version 25.0.0.1) for Mac. Mann–Whitney and Kruskal–Wallis tests were used for the analysis of continuous variables. Chi-square and Fisher’s exact tests were used in comparison of categorical variables. Logistic regression analysis was used to analyze the key outcome variables (use and number of RBC transfusions, time to surgery, mobilized before discharge) with adjustment for the number of comorbidities, use of walking aids, need for assistance in daily activities, and presence of memory disorder (diagnosed dementia or recorded memory problems without a diagnosis). Cox regression was used for similar adjusted analyses concerning the time to mobilization. The results of the regression analyses are reported as odds/hazard ratios with 95% confidence intervals.

### Statement of ethics

Approval from ethics committee or informed consent was not required for this registry-based study. The use of patient records was authorized by the hospital according to local guidelines (R18568S).

## Results

In total, 153 cases in 151 patients, and 153 controls were included, representing 53% of all hip fracture operations during the study period. The cases and the controls were comparable in terms of age, gender, living arrangements, and prevalence of memory disorders (Table 1). Their median age was 87 years, almost two-thirds were women, and > 70% lived at home before the fracture. The cases had on average one chronic disease (4 vs 3; *p* < 0.001) and two regular medications (8 vs 6; *p* < 0.001) more than the controls. The controls seemed to have better functional ability (based on use of walking aids) and independence in daily activities (Table 1). Thromboembolic risk of the cases was slightly higher than controls in terms of CHA_2_DS_2_-VASc score, while the risks of bleeding were similar (Table 1). There were no statistically significant differences in fracture types.

Majority of cases (*n* = 132, 86%) used antithrombotic medication due to atrial fibrillation. Other reasons were pulmonary embolism or deep venous thrombosis (*n* = 9, 6%), history of stroke (*n* = 13, 9%), coronary artery disease (*n* = 3, 2%), and mechanical heart valve (*n* = 3, 2%). Some patients had multiple reasons for antithrombotic use. The antithrombotic used was usually warfarin (*n* = 115, 75%), followed by DOACs (apixaban *n* = 8, 5%, rivaroxaban *n* = 13, 9%). ADP inhibitors were used by 10 patients (clopidogrel, *n* = 9, 6%; ticagrelor *n* = 1, 1%).

### Association with outcomes

Cases were operated more often in general anesthesia than controls (Table 2). TTS and duration of surgery were similar in both groups and there was no difference in blood loss, but the time spent at PACU was longer in cases (276 vs. 231 min, *p* < 0.001). Cases received more RBC transfusions than controls, especially the percentage of patients who received ≥ 2 RBC units was higher (16% vs 5%, *p* = 0.007). There were no differences in hemoglobin levels.

**Table 2:** Perioperative and post-operative characteristics of cases and controls

	Cases (*n* = 153)	Controls (*n* = 153)	*P* value
Type of surgery			0.277
Hemiarthroplasty	49%	60%	
Intramedullary nailing	46%	36%	
Other	5%	4%	
Time from admission to surgery, days	1* (0–7)	1* (0–4)	0.755
General anesthesia	16%	7%	0.007
Duration of surgery, minutes (*n* = 299)	72* (28–279)	69* (11–389)	0.138
Time from beginning of operation until discharge from PACU, minutes** (*n* = 294)	276* (115–1105)	231* (116–912)	< 0.001
Blood loss (mL)	250* (30–5500)	230* (10–1750)	0.187
Number of transfused RBC units (*n* = 295)			0.007
None	74%	86%	
1	10%	9%	
≥ 2	16%	5%	
Lowest hemoglobin during treatment (g/L)	100 (67–135)	101 (68–148)	0.458
Decrease of hemoglobin during hospitalization (g/L)	22 (0–82)	22 (0–47)	0.830
Postoperative day of mobilization*** (*n* = 113)	2* (1–17)	1* (1–6)	0.004
Mobilized before discharge from the operating hospital	18%	29%	0.015
Postoperative day of IUC removal (*n* = 67)	6* (0–28)	4* (0–11)	0.163
IUC removed before discharge from the operating hospital	5%	6%	0.608
Length of stay at the operating hospital	3* (0–10)	3* (1–12)	0.151
Total length of stay*** (*n* = 52)	17* (10–35)	14* (3–35)	0.057
30-day mortality	11%	6%	0.101

^*^Median (range), **post-anesthesia care unit, ***available for patients mobilized at the operating hospital or discharged to the orthogeriatric ward of the largest city in the area

Postoperatively controls were mobilized one day earlier (on the 2nd vs. the 1st post-operative day, *p* = 0.004) and a higher percentage of the controls were mobilized before discharge from the operating hospital (29% vs. 18% in cases, *p* = 0.015). IUC was usually removed in secondary care unit with both cases and controls and there was no difference in the time to IUC removal. Among patients who were followed also at the secondary care ward, the duration of treatment tended to be longer in cases than in controls (Table 2). There was no difference in the 30-day mortality.

### Comparison between different antithrombotics

Users of DOACs and ADP inhibitors were operated later and more often under general anesthesia than warfarin users and controls (Table 3). Furthermore, the number of RBC transfusions during the hospital stay was higher in all antithrombotic groups: DOACs received ≥ 2 units of RBCs over 5 times more likely than controls (Table 3). Users of any type of antithrombotics spent more time in PACU than controls, especially in DOAC and ADP inhibitor groups (Table 3). Post-operative mobilization happened one day later for warfarin group and two days later in the DOAC and ADP inhibitor groups than in controls (on 2nd, 3rd, and 3rd compared to 1st post-operative day, *p* = 0.013).

There were no statistically significant differences between the groups in duration of surgery, blood loss, the lowest level of hemoglobin or changes in hemoglobin during hospitalization, proportion of patients mobilized and having IUC removed by discharge from the orthopedic ward, and total length of stay or 30-day mortality (Table 3).

**Table 3:** Perioperative and post-operative characteristics of patients using different types of antithrombotics and controls

	Warfarin (*n* = 115)	DOACs (*n* = 28)	ADP inhibitors (*n* = 10)	Controls (*n* = 153)	*P* value
Time from admission to surgery, days	1* (0–5)	2* (1–3)	2.5* (1–7)	1* (0–4)	< 0.001
General anesthesia	10%	18%	90%	7%	< 0.001
Duration of surgery, minutes (*n* = 299)	72* (28–279)	68* (35–211)	87* (31–129)	69* (11–389)	0.417
Time from beginning of operation until discharge from PACU, minutes** (*n* = 294)	268* (137–1105)	290* (136–477)	285* (159–353)	231* (116–912)	0.003
Blood loss (mL)	250* (30–5500)	210* (50–2050)	375* (100–650)	230* (10–1750)	0.201
Number of transfused RBC units (*n* = 295)					0.003
None	78%	63%	60%	86%	
1	8%	11%	30%	9%	
≥ 2	14%	26%	10%	5%	
Lowest level of hemoglobin during treatment (g/L)	101* (68–135)	95* (67–117)	103* (87–119)	101* (68–148)	0.515
Decrease in hemoglobin during hospitalization (g/L)	22* (0–57)	22* (0–82)	21* (5–44)	22* (0–47)	0.994
Postoperative day of mobilization*** (*n* = 113)	2* (1–17)	3* (3–11)	3* (2–5)	1* (1–6)	0.013
Mobilized before discharge from the operating hospital	19%	14%	10%	29%	0.089
Postoperative day of IUC removal (*n* = 67)	4* (0–28)	8.5* (4–17)	3.5* (1–6)	4* (0–11)	0.139
IUC removed before discharge from the operating hospital	5%	0%	10%	6%	0.545
Length of stay at the operating hospital	3* (0–8)	4* (2–7)	3,5* (2–10)	3* (1–12)	0.001
Total length of stay*** (*n* = 52)	14.5* (10–32)	19.5* (19–35)	-	14* (3–35)	0.084
30-day mortality	10%	18%	10%	6%	0.153

^*^Median (range), **post-anesthesia care unit, ***available for patients mobilized at the operating hospital or discharged to the orthogeriatric ward of the largest city in the area

### Adjusted analyses

After adjustments, antithrombotic use was associated with receiving ≥ 2 units of RBCs (adjusted OR 3.00, 95% CI 1.03–8.72). DOACs were associated with delayed surgery (adjusted OR 2.95, 95% CI 1.11–7.85) and receiving for ≥ 2 units of RBCs (adjusted OR 7.02, 95% CI 1.73–28.54).

**Fig. 1:**
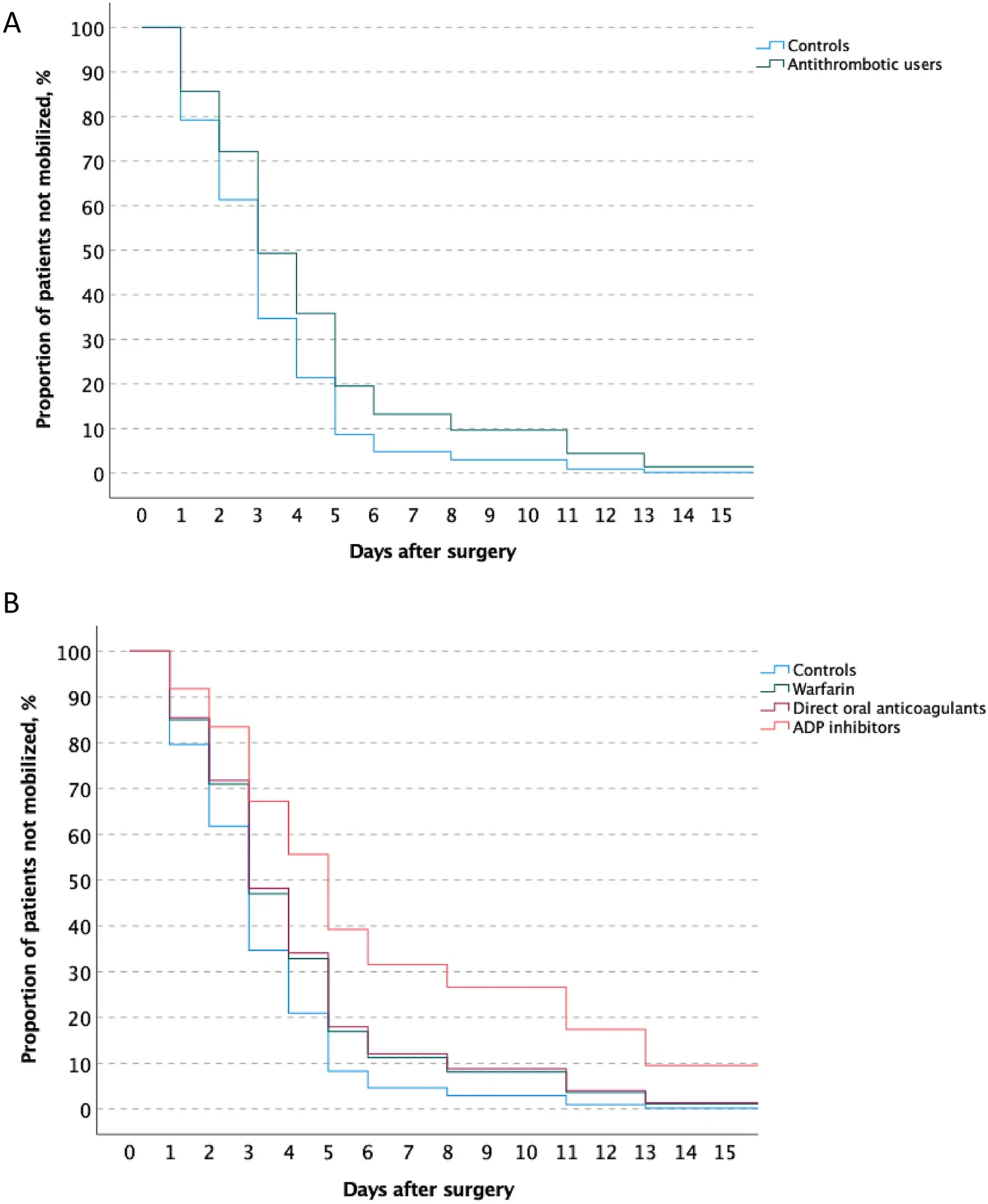
Modeled probability of mobilization according to antithrombotic use (**A**) and type of antithrombotic (**B**), based on Cox regression analysis adjusted for the number of comorbidities, use of walking aids, need for assistance in daily activities and presence of memory disorder

Adjustment leveled the difference in mobilization between cases and controls (Fig. 1A, Table 4), but the tendency of lower rate of mobilization in association with antithrombotic use remained. A similar result was observed for warfarin and DOACs, whereas in the users of ADP inhibitors, the hazard of mobilization seemed to be lower—although not statistically significantly—and mobilization to be delayed in comparison to controls.

**Table 4:** Time to and probability of mobilization in patients using antithrombotics and their controls

Antithrombotic use	Mobilized during available follow-up *n* (%)	Postoperative day of mobilization, median	Cox regression analysis with mobilization as the endpoint					
			HR	95% CI	*p*	Adjusted* HR	95% CI	*p*
Cases	51/153 (33%)	2	0.58	0.39–0.85	0.005	0.67	0.41–1.08	0.098
Controls	62/153 (41%)	1	1			1		
Warfarin	38/115 (33%)	2	0.60	0.39–0.91	0.015	0.71	0.42–1.20	0.20
DOAC	10/28 (35%)	3	0.52	0.26–1.03	0.062	0.69	0.33–1.43	0.319
ADP inhibitors	3/10 (30%)	3	0.56	0.17–1.71	0.291	0.38	0.11–1.28	0.117
Controls	62/153 (41%)	1	1			1		

^*^Adjustment for the number of comorbidities, use of walking aids, need for assistance in daily activities, and presence of memory disorder

In other outcomes, there were no statistically significant differences, partly because of insufficient number of patients.

## Discussion

Antithrombotics were associated with later mobilization and more RBC transfusions. DOACs were associated with delayed surgery and need of plural (2 or more) units of RBCs also after adjustments. Patients using DOACs or ADP inhibitors got operated later and were transferred later from operating hospital in comparison to controls and warfarin users. More importantly, the results suggest that antithrombotic use is associated with later mobilization. These observations support our hypothesis that antithrombotic use leads to delays in care and recovery both before and after surgery. However, after adjustments, some of the differences were not statistically significant due to insufficient statistical power.

Our finding regarding later operation time and DOAC and ADP inhibitor use is in line with previous studies and meta-analyses [11, 13–15, 21–24], and the association remained after adjustments. Several earlier studies suggest that warfarin use is associated with a shorter delay to surgery than DOAC and ADP inhibitor use [9, 11, 15], while we did not observe delay to surgery in warfarin users. This difference might be explained with active cooperation of emergency department and operating surgeons or due to planned protocols for reversal of warfarin with vitamin K or prothrombin complex concentrate [25]. Such reversal protocols have been shown to halve the delay to surgery [22]. In the case of DOACs and ADP inhibitors, comparable ways to reverse their effect are not available (except for dabigatran) and there are also difficulties in monitoring the effects and quantities of these drugs in blood. Aiming at early surgery would however be important in hip fracture patients as a systematic review and meta-analysis concluded that surgery in 48 h is associated with 20% lower risk of dying within 1-year interval and fewer post-operative complications [4]. In our series, a greater proportion of patients using antithrombotics than controls was operated in general anesthesia. Increased use of general anesthesia could be a way to reduce the adverse effects of delayed surgery on DOAC and ADP inhibitor users [26], according to a recent RCT indicating that general and spinal anesthesia result in similar mortality, mobility, and delirium risk following hip fracture [27].

Our findings regarding antithrombotic and DOAC use and the need for RBC transfusions are mostly in line with previous findings [10, 16, 28], while some studies report no association [15, 24]. In a large Danish nationwide cohort study where results were adjusted for multiple variables (e.g., age, sex, surgical delay), DOACs were associated with increased risk of RBC transfusions [16], but it was not reported how many units of RBCs were given. Our study adds to previous literature a finding that even after adjustments both antithrombotics and DOACs are associated with a need for plural units of RBCs. On the other hand, no differences in hemoglobin levels or blood loss in the operation were noted. Mixed results may relate to differences in transfusion thresholds or patient characteristics like comorbidity. Timing of surgery may also play a role: some studies suggest that early surgery in ADP inhibitor and DOAC users is associated with more anemia and transfusions [8, 17]. This may be related both to increased hematoma formation particularly when surgery is delayed and increased bleeding in association with surgery. The relevance of the elevated risk of RBC transfusions is not yet clear. Post-operative RBC transfusions have been linked with higher mortality, post-operative morbidity, longer stay in hospital, and higher hospital admission rate [29]. However, no association has been found with warfarin or DOAC use and 30-day mortality in this or earlier studies [28].

The key finding of our study was that antithrombotic users were mobilized on average post-operatively at least one day later than controls. In addition to mobilizing later, DOAC and ADP inhibitor subgroups had their surgery done later than controls, making the total period of immobility even longer. After adjustments, mobilization among warfarin and DOAC users was similar to that of controls, whereas the difference between users of ADP inhibitors and controls persisted and in fact appeared more pronounced. Due to small amount of ADP inhibitor users, the difference was however not statistically significant and warrants confirmation in larger series.

To the best of our knowledge, antithrombotic use has not been associated with later mobilization previously. The finding is worrying when coupled with longer time to surgery as among older people with possibly limited physical activity even a small loss of muscle mass during the immobilization can lead to major decline of functional capacity [30]. This could lead to longer length of stay and higher risk of institutionalization and adversely affect long-term quality of life. Indeed, post-operative sarcopenia has been associated with poorer mobility and greater rate of nursing home admissions one year after hip fracture [31]. Moreover, early mobilization has been associated with lower in-hospital and 30-day mortality [6, 32, 33]. The mechanisms underlying delayed mobilization remain unclear but potentially include acute sarcopenia, nutritional issues (fasting before surgery), need for RBC transfusions, and wound complications. It is also possible that besides antithrombotic use, comorbidities affect post-operative mobilization. However, because comorbidity may also complicate recovery, it is essential to eliminate modifiable system-related factors that could further worsen the situation.

Since late 2010s, several guidelines and consensus statements have been published regarding hip fracture surgery in patients using Antithrombotics [34–36]. The message of the guidelines strongly supports early surgery within 24–48 h and even with spinal anesthesia, depending on risk–benefit assessment (drug concentrations, renal function, comorbidity) also in patients using DOACs and ADP inhibitors. Later studies give further support to the guidelines, showing that early surgery in such patients is associated with shorter length of stay and mortality despite greater need for transfusions [8, 17, 22], emphasizing the importance of avoiding delays and effects of early mobilization.

The strength of our study is the comprehensive data of our population-based study, including essential baseline information, such as functional ability, chronic diseases, living arrangements, perioperative care, and early post-operative phase. The comprehensive data set allowed adjusting for several background variables often ignored in previous studies. However, due to the retrospective study design, patient- or provider-related reasons for surgical delays could not be considered. Unfortunately, the relatively small study population precluded a thorough analysis of the DOAC and ADP inhibitor groups and complicated the adjusted analyses. The proportion of DOAC users was also relatively small as they were introduced later in Finland than in some other countries. For these reasons, the results concerning DOACs and ADP inhibitors must be considered preliminary, and the possibility for residual confounding should be kept in mind. Furthermore, we were able to follow up recovery after discharge from orthopedic ward only in patients treated in the adjacent secondary health care unit; otherwise the data were limited to the first post-operative days. Calculating TTS and time to mobilization in hours might have improved detection in differences across the patient groups but was not feasible. Selection of controls was suboptimal, but hardly biases the results. It is unlikely that the difference in the date of surgery affects the studied outcomes, and the fracture types did not differ significantly. The type of surgery may affect recovery, but it was not considered in the analyses. However, most fractures of the same type are treated in the same way, and there were no patients aged < 70 years (who might be treated with osteosynthesis rather than hemiarthroplasty) and no total hip replacements (that are performed in a separate hospital in our area). Finally, the data were collected in late 2010s, and after that, DOACs have largely replaced warfarin in the treatment of atrial fibrillation. However, a considerable proportion of older patients continues to use warfarin [37, 38] and hence a certain share of hip fracture patients uses it [23, 24]. Moreover, despite the existing guidelines, delays to surgery remain in anticoagulant users [23, 24], making our study still clinically relevant.

## Conclusions

The use of antithrombotics increases time to surgery and may delay post-operative mobilization and hence adversely affect post-operative recovery. Together with existing evidence, this study underlines the importance of undelayed surgery and early mobilization in patients using antithrombotics to achieve the desired outcomes.

## Data Availability

The datasets used in this study are not available as the use of the data was approved only for the purposes of the this study.

## References

[1] Dyer SM, Crotty M, Fairhall N, Magaziner J, Beaupre LA, Cameron ID et al (2016) A critical review of the long-term disability outcomes following hip fracture. BMC Geriatr 16(1):58 10.1186/s12877-016-0332-0 PMC5010762 27590604

[2] Pajulammi HM, Pihlajamäki HK, Luukkaala TH, Nuotio MS (2015) Pre- and perioperative predictors of changes in mobility and living arrangements after hip fracture—A population-based study. Arch Gerontol Geriatr 61:182–189 10.1016/j.archger.2015.05.007 26043958

[3] Leer-Salvesen S, Engesæter LB, Dybvik E, Furnes O, Kristensen TB, Gjertsen JE (2019) Does time from fracture to surgery affect mortality and intraoperative medical complications for hip fracture patients? An observational study of 73 557 patients reported to the Norwegian hip fracture register. Bone Joint J. 10.1302/0301-620x.101b9.bjj-2019-0295.r1 31474142

[4] Klestil T, Röder C, Stotter C, Winkler B, Nehrer S, Lutz M et al (2018) Impact of timing of surgery in elderly hip fracture patients: a systematic review and meta-analysis. Sci Rep 8:1–15 10.1038/s41598-018-32098-7 PMC6141544 30224765

[5] Su B, Newson R, Soljak H, Soljak M (2018) Associations between post-operative rehabilitation of hip fracture and outcomes: national database analysis. BMC Musculoskelet Disord 19:1–9 10.1186/s12891-018-2194-4 PMC6145207 30231867

[6] Agarwal N, Feng T, Maclullich A, Duckworth A, Clement N (2024) Early mobilisation after hip fracture surgery is associated with improved patient outcomes: a systematic review and meta‐analysis. Musculoskelet Care. 10.1002/msc.1863 38353351

[7] Taranu R, Redclift C, Williams P, Diament M, Tate A, Maddox J et al (2018) Use of Anticoagulants Remains a Significant Threat to Timely Hip Fracture Surgery. Geriatr Orthop Surg Rehabil 9:215145931876415 10.1177/2151459318764150 PMC5871052 29619276

[8] Yang Z, Ni J, Long Z, Kuang L, Gao Y, Tao S (2020) Is hip fracture surgery safe for patients on antiplatelet drugs and is it necessary to delay surgery? A systematic review and meta-analysis. J Orthop Surg Res 15:105 10.1186/s13018-020-01624-7 PMC7068917 32164755

[9] Lawrence JE, Fountain DM, Cundall-Curry DJ, Carrothers AD (2017) Do patients taking warfarin experience delays to theatre, longer hospital stay, and poorer survival after hip fracture? Clin Orthop Relat Res 475:273–279 10.1007/s11999-016-5056-0 PMC5174047 27586655

[10] Bruckbauer M, Prexl O, Voelckel W, Ziegler B, Grottke O, Maegele M et al (2019) Impact of direct oral anticoagulants in patients with hip fractures. J Orthop Trauma 33:E8-13 10.1097/BOT.0000000000001329 30277979

[11] Cafaro T, Simard C, Tagalakis V, Koolian M (2019) Delayed time to emergency hip surgery in patients taking oral anticoagulants. Thromb Res 184:110–114 10.1016/j.thromres.2019.11.005 31722273

[12] Leer-Salvesen S, Dybvik E, Ranhoff AH, Husebø BL, Dahl OE, Engesæter LB et al (2020) Do direct oral anticoagulants (DOACs) cause delayed surgery, longer length of hospital stay, and poorer outcome for hip fracture patients? Eur Geriatr Med 11:563–569 10.1007/s41999-020-00319-w PMC7438280 32361891

[13] López S, da Casa C, Pablos-Hernández C, Pescador D, Díaz-Álvarez A, Asensio N et al (2020) The impact of antithrombotic therapy on surgical delay and 2-year mortality in older patients with hip fracture: a prospective observational study. Eur Geriatr Med 11:555–561 10.1007/s41999-020-00293-3 32297256

[14] You D, Xu Y, Ponich B, Ronksley P, Skeith L, Korley R et al (2021) Effect of oral anticoagulant use on surgical delay and mortality in hip fracture. Bone Joint J 103-B(2):222–233 10.1302/0301-620X.103B2.BJJ-2020-0583.R2 33517730

[15] Baldino JB, Bellas NJ, Solomito MJ, Kumar M, Davis SL (2025) Direct oral anticoagulants (DOACs) increase time to operating room without increasing postoperative hematologic complications in patients with fragility fractures of the proximal femur. Injury 56:112217 10.1016/j.injury.2025.112217 39983534

[16] Daugaard C, Pedersen AB, Kristensen NR, Johnsen SP (2019) Preoperative antithrombotic therapy and risk of blood transfusion and mortality following hip fracture surgery: a Danish nationwide cohort study. Osteoporosis Int 30:583–591 10.1007/s00198-018-4786-0 30498889

[17] Brameier DT, Tischler EH, Ottesen TD, McTague MF, Appleton PT, Harris MB et al (2024) Use of direct oral anticoagulants among patients with hip fracture is not an indication to delay surgical intervention. J Orthop Trauma 38:148–154 10.1097/BOT.0000000000002753 38385974

[18] Buecking B, Bohl K, Eschbach D, Bliemel C, Aigner R, Balzer-Geldsetzer M et al (2015) Factors influencing the progress of mobilization in hip fracture patients during the early postsurgical period?-A prospective observational study. Arch Gerontol Geriatr 60:457–463 10.1016/j.archger.2015.01.017 25682536

[19] Said CM, Delahunt M, Ciavarella V, al Maliki D, Boys A-M, Vogrin S et al (2021) Factors impacting early mobilization following hip fracture: an observational study. J Geriatr Phys Ther 44:88–93 10.1519/JPT.0000000000000284 33534334

[20] Borre ED, Goode A, Raitz G, Shah B, Lowenstern A, Chatterjee R et al (2018) Predicting thromboembolic and bleeding event risk in patients with non-valvular atrial fibrillation: a systematic review. Thromb Haemost 118:2171–2187 10.1055/s-0038-1675400 PMC6754740 30376678

[21] Frenkel Rutenberg T, Velkes S, Vitenberg M, Leader A, Halavy Y, Raanani P et al (2018) Morbidity and mortality after fragility hip fracture surgery in patients receiving vitamin K antagonists and direct oral anticoagulants. Thromb Res 166:106–112 10.1016/j.thromres.2018.04.022 29727737

[22] You D, Xu Y, Krzyzaniak H, Korley R, Carrier M, Schneider P (2023) Safety of expedited-surgery protocols in anticoagulant-treated patients with hip fracture: a systematic review and meta-analysis. Can J Surg 66:E170–E180 10.1503/cjs.010021 PMC10069416 37001973

[23] Wijekulasuriya S, Seymour H, Tarrant S, Balogh ZJ, Navarre P, Hallen J et al (2025) The impact of direct oral anticoagulants on hip fracture care in Australia and New Zealand: a prospective patient sprint audit. Osteoporosis Int 36:2137–2147 10.1007/s00198-025-07659-y PMC12628422 40828478

[24] Farhan-Alanie MM, Chinweze R, Walker R, Eardley WGP (2024) The impact of anticoagulant medications on fragility femur fracture care: the hip and femoral fracture anticoagulation surgical timing evaluation (HASTE) study. Injury 55:111451 10.1016/j.injury.2024.111451 38507942

[25] Chai-Adisaksopha C, Hillis C, Siegal DM, Movilla R, Heddle N, Iorio A et al (2016) Prothrombin complex concentrates versus fresh frozen plasma for warfarin reversal: a systematic review and meta-analysis. Thromb Haemost 116:879–890 10.1160/TH16-04-0266 27488143

[26] Shah R, Sheikh N, Mangwani J, Morgan N, Khairandish H (2021) Direct oral anticoagulants (DOACs) and neck of femur fractures: standardising the perioperative management and time to surgery. J Clin Orthop Trauma 12:138–147 10.1016/j.jcot.2020.08.005 PMC7920209 33716439

[27] Neuman MD, Feng R, Carson JL, Gaskins LJ, Dillane D, Sessler DI et al (2021) Spinal anesthesia or general anesthesia for hip surgery in older adults. N Engl J Med 385:2025–2035 10.1056/NEJMoa2113514 34623788

[28] Cheung ZB, Xiao R, Forsh DA (2021) Time to surgery and complications in hip fracture patients on novel oral anticoagulants: a systematic review. Arch Orthop Trauma Surg 142:633–640 10.1007/s00402-020-03701-2 33417028

[29] Arshi A, Lai WC, Iglesias BC, McPherson EJ, Zeegen EN, Stavrakis AI et al (2020) Blood transfusion rates and predictors following geriatric hip fracture surgery. Hip Int 31:272–279 10.1177/1120700019897878 31912747

[30] Wall BT, Dirks ML, van Loon LJC (2013) Skeletal muscle atrophy during short-term disuse: Implications for age-related sarcopenia. Ageing Res Rev 12:898–906 10.1016/j.arr.2013.07.003 23948422

[31] Steihaug OM, Gjesdal CG, Bogen B, Kristoffersen MH, Lien G, Hufthammer KO et al (2018) Does sarcopenia predict change in mobility after hip fracture? A multicenter observational study with one-year follow-up. BMC Geriatr 18:65 10.1186/s12877-018-0755-x PMC5836393 29506481

[32] Ferris H, Brent L, Coughlan T (2020) Early mobilisation reduces the risk of in-hospital mortality following hip fracture. Eur Geriatr Med 11:527–533 10.1007/s41999-020-00317-y 32297275

[33] Goubar A, Martin FC, Potter C, Jones GD, Sackley C, Ayis S et al (2021) The 30- day survival and recovery after hip fracture by timing of mobilization and dementia. Bone Joint J 103:1317–1324 10.1302/0301-620X.103B7.BJJ-2020-2349.R1 PMC7611209 34192935

[34] Griffiths R, Babu S, Dixon P, Freeman N, Hurford D, Kelleher E et al (2021) Guideline for the management of hip fractures 2020: guideline by the association of anaesthetists. Anaesthesia 76:225–237 10.1111/anae.15291 33289066

[35] Mitchell RJ, Wijekulasuriya S, Mayor A, Borges FK, Tonelli AC, Ahn J et al (2024) Principles for management of hip fracture for older adults taking direct oral anticoagulants: an international consensus statement. Anaesthesia 79:627–637 10.1111/anae.16226 38319797

[36] Kearns R, Jenkins P, Bagot C. Clinical guideline. Anticoagulation management in patients admitted to orthopaedics with hip fractures. NHS Scotland. Available online: https://www.rightdecisions.scot.nhs.uk/ggc-clinical-guidelines/anaesthesia/patient-care/external-drug-management/anticoagulation-management-in-patients-admitted-to-orthopaedics-with-hip-fractures-424/ Accessed Jan 15 2026

[37] Pilotto A, Veronese N, Polidori MC, Strandberg T, Topinkova E, Cruz-Jentoft AJ et al (2023) Frailty and anticoagulants in older subjects with atrial fibrillation: the EUROSAF study. Age Ageing 52:afad216 10.1093/ageing/afad216 PMC11373113 39248098

[38] Williams BA, Blankenship JC, Voyce S, Chang AR (2024) Trends over time in oral anticoagulation and stroke rates in atrial fibrillation: a community-based study. J Stroke Cerebrovasc Dis 33:108081 10.1016/j.jstrokecerebrovasdis.2024.108081 39396659

